# Phytochemical Analysis, Ephedra Procera C. A. Mey. Mediated Green Synthesis of Silver Nanoparticles, Their Cytotoxic and Antimicrobial Potentials

**DOI:** 10.3390/medicina55070369

**Published:** 2019-07-12

**Authors:** Muhammad Qasim Nasar, Ali Talha Khalil, Muhammad Ali, Mehwish Shah, Muhammad Ayaz, Zabta Khan Shinwari

**Affiliations:** 1 Department of Biotechnology, Quaid-i-Azam University, Islamabad 45320, Pakistan; 2 Department of Eastern Medicine and Surgery, Qarshi University, Lahore 54000, Pakistan; 3 State Key Laboratory of Chemo/Biosensing and Chemometrics, Collage of Chemistry and Chemical Engineering, Hunan University, Changsha 410082, China; 4 Departant of Pharmacy, University of Malakand, Khyber Pakhtunkhwa 18800, Pakistan; 5 Lahore campus, Qarshi University, Lahore 54000, Pakistan; 6 Pakistan Academy of Sciences, Islamabad 44000, Pakistan

**Keywords:** *Ephedra procera*, green chemistry, microbial resistance, HepG2 cells, hemolysis, nanotechnology

## Abstract

*Background and Objectives:* The current study focuses on an eco-friendly and cost-effective method of *Ephedra procera* C. A. Mey. mediated green synthesis of silver nanoparticles as potential cytotoxic, antimicrobial and anti-oxidant agents. *Materials and Methods:* Plant aqueous extracts were screened for Total Phenolic (TPC), Total Flavonoid contents (TFC), Total Antioxidant Capacity (TAC) and 2,2-diphenyl-1-picrylhydrazyl (DPPH) free radical scavenging potentials. Total reducing power estimated by potassium ferricyanide colorimetric assay. The biosynthesized *E. procera* nanoparticles (EpNPs) were characterized by UV-spectroscopy, Fourier-transform infrared spectroscopy (FTIR), X-ray diffraction and Scanning electron microscopy. EpNPs were evaluated for their antimicrobial, bio-compatibility and cytotoxic potentials. *Results:* Initial phytocheimcal analysis of plant aqueous extract revealed TFC of 20.7 ± 0.21 µg/mg extract and TPC of 117.01 ± 0.78 µg/mg extract. TAC, DPPH free radical scavenging and reducing power were 73.8 ± 0.32 µg/mg extract, 71.8 ± 0.73% and 105.4 ± 0.65 µg/mg extract respectively. The synthesized EpNPs were observed to possess high cytotoxicity against HepG2 cancer cell lines with IC_50_ (61.3 µg/mL) as compared aqueous extract with IC_50_ of (247 µg/mL). EpNPs were found to be biocompatible and have less effect on human erythrocytes. EpNPs exhibited significant antioxidant potentials and exhibited considerable activity against *Escherichia coli* and *Bacillus subtilis* with Minimum Inhibitory Concentration (MICs) of 11.12 μg/mL and 11.33 μg/mL respectively. Fungal species *Aspergillus niger* and *Aspergillus flavus* were found susceptible to EpNPs. *Conclusions:* Results of the current study revealed that EpNPs exhibited considerable antibacterial, antifungal and cytotoxic potentials. Aqueous extract possesses significant anti-radical properties and thus can be useful in free radicals induced degenerative disorders.

## 1. Introduction

Nature sanctified the anthropoid with variety of medications to cure numerous maladies [1,2]. Most of the human populace is still consuming plant-based prescriptions [3,4,5]. In the developing countries, it is assessed that about 95% of rural and 70% of urban births are assisted with the use of traditional medicines as pre- and post-maternity cares [6]. Nanotechnology is an emerging feat in sciences that involves processing of materials at nanoscale (1–100 nm) in a controlled manner so that their unique properties can be utilized in different myriad applications [7,8]. Plants provide an exciting interface for the synthesis of nanoparticles but must be looked under the lens of “No Net Loss”. The exploration of medicinal plants for synthesis purposes needs to be counter balanced with the cultivation of the same plants [9]. Nobel metal nanoparticles have gained popularity because of their distinctive features [10,11]. A recent application of medicinal plants involves green synthesis of metal nanoparticles using plant extracts as reducing agents [12,13,14]. The synthesis of nanoparticles with the help of plant extracts offers an inclusive array of advantages over other biological synthesis as a plant extract does not require the maintenance of bacterial, fungal and algal cultures [15,16]. Sastry and his coworkers are pioneers in synthesis of nanoparticle by using plant extracts [17,18]. This eco-friendly method is more biocompatible that have capability at larger synthesis [19]. Approaches of nanoparticle production through various physical and chemical ways have their own shortcomings as they produce massive environmental pollutions and lethal side effects. Consequently, there is a prerequisite for “green chemistry” that certifies hygienic, non-toxic, and eco-friendly methods [19,20]. The cumulative ultimatum for functionalized nanoparticles has fortified new developing bio-routes. Synthesis of nanoparticles by using plant extracts as reducing, capping and oxidizing agents has received distinct consideration among others [21]. Hence, medicinal plants having well-known therapeutic prominence are extensively used for the shape and size controlled synthesis of silver nanoparticles [22,23,24]. Among other metals, silver nanoparticles (AgNPs) have received substantial focus due to their better stability, excellent catalytic potential and good electrical conductivity. Silver more effectively react with sulfur of the proteins present in biological membranes and phosphorus present in DNA. The benefits of using plants for the production of nanoparticles include their accessibility, care in handling, and existence of an inconsistency of metabolites that may assistance in reducing silver. The size of nanoparticles is effected by varying key factors such as temperature, pH, substrate concentration, and time of exposure to the substrate [25].

Plant extracts contain secondary metabolites such as phenols, flavonoids, and terpenoids which are implicated in bio-reduction of metal into NPs [13,26]. In detail, in regards to the underlying mechanism of bio-reduction of silver, it was assumed that first Ag^+^ ions are attached on the surface of proteins present in extract through electrostatic interactions and then reduced by them, leading to alterations in their secondary structure and the formation of silver nuclei. Afterward silver nuclei propagate by the further reduction of silver ions and their accumulation [27]. *Ephedra major* subsp. procera (C. A. Mey.) Bornm (Synonym: *Ephedra procera)* belongs to family Ephedraceae that has only genus Ephedra, which consists of about 45 species of perennials and shrubs [28]. The genus Ephedra is a source of different medicinal phytochemicals [29]. Medicinally important compounds such as flavano-flavonol ephedrannin, Ephedroxane, Cyclopropyl-A amino acids, Flavones, Flavanols, Bisflavanolsuch and carboxylic acids have been isolated from different parts of *Ephedra* species [29,30,31]. The genus Ephedra consists of species which show antibacterial potential [32,33]. To the best of our knowledge, this is the first research work on *E. procera* aqueous extract mediated optimized green synthesis of AgNPs and evaluation of their antimicrobial, Bio-compatibility and anticancer potential.

## 2. Materials and Methods

### 2.1. Drugs and Chemicals

Dipotassium hydrogen phosphate, potassium dihydrogen phosphate, ferric chloride, trichloroacetic acid, ammonium molybdate, potassium ferricyanide, sulphuric acid, aluminium chloride, gallic acid, quercetin, ascorbic acid, potassium acetate, were obtained from Merck, Darmstadt, Germany, The Tween-20 was bought from (Merck), DPPH, Folin-Ciocalteu reagent (FCR) and were bought from (Sigma-Aldrich, St. Louis, MO, USA) while phosphate buffer saline (PBS), nutrient agar, Sabouraud dextrose (SDA), trypton soy broth (TSB), sterile normal saline solution (0.9%), sea salt, 0.5% triton x-100, cefixime were obtained from Sigma (Sigma-Aldrich, St. Louis, MO, USA) and amphotericin-B form Caisson.

### 2.2. Cultures and Cell Lines

Fungal strains including *A. flavus* (FCBP 0064)*, A. fumigatus* (FCBP 66), *A. niger* (FCBP 0198) and *Mucor* specie (FCBP 0300) were obtained from fungal culture bank of Pakistan. The bacterial strains used for antibacterial assay were (*K. pneumoniae* ATCC-1705), (*S. aureus* ATCC-6538), (*P. aeruginosa* ATCC-9721), (*S. epidermidis* ATCC-12228), (*E. coli* ATCC-25922) and (*B. subtilis* ATCC-6633). HepG2 cancer cell lines (RBRC-RCB1648) were used to determine the anticancer potential and fresh human blood used for haemolytic activity was obtained from volunteered lab fellow and used after his permission.

### 2.3. Collection of Plant Sample

Fresh aerial parts of *E. procera* was collected in April 2016 from kawas village of District Ziarat, Balochistan, Pakistan. Plant was identified by Professor Dr. Zabta Khan Shinwari and herbarium sheet under voucher No. 349 was deposited at Department of Biotechnology, Quaid-i-Azam University Islamabad, Pakistan.

#### Extract Preparation

Plant was shade dried and ground into fine powder. Powder (10 g) was boiled in 200 mL of autoclaved distilled H_2_O for 10 min. After boiling, it was cooled down, filtered, stored at 4 °C in refrigerator and further used for phytochemical analysis and nanoparticles synthesis [34,35].

### 2.4. Phytochemical Analysis of the Extract

#### 2.4.1. Total Phenolic Content (TPC)

TPC of *E. intermedia* aqueous extract was appraised following our previously reported method [36]. Briefly, 1 mL from various concentrations of plant aqueous extract was added to nine ml of double ionized distilled water. Following this, 1 ml of FCR reagent was added to the mixture and shacked vigorously. To the mixture, 10 mL of 7% Na_2_CO_3_ solution was added followed by mixing. Further, 25 mL of Distilled water was added to the mixture and absorbance was measured at 750 nm via UV spectrophotometer. Gallic acid (GA) standard curve was used quantify TPC and was expressed in mg equivalent of GA.

#### 2.4.2. Total Flavonoid Content (TFC)

In TFC analysis, plant extract (0.3 mL) was mixed with 0.15 mL of 0.5M NaNO_2_, 3.4 mL of 30 percent methanol, 015 mL of 0.3M AlCL_3_.6H_2_O and mixed. To the mixture, 1 mL of 1 M NaOH solution was added after 5 min. Absorption was measured at 506 nm via UV spectrophotometer. Rutin standard curve was from pure rutin solution and TFC were calculated from rutin standard curve and expressed as mg of rutin [37].

#### 2.4.3. Total Antioxidant Capacity

Using phosphomolybdenum method the antioxidant capacity of *E. intermedia* aqueous extract was elucidated. In brief, 1 mL of reagents mixture consisting of 0.6 M H_2_SO_4_, 4 mM (NH_4_)_6_ MO_7_O_24_.4H_2_O and 28 mM NaPO_4_ was mixed with 100 µL of plant sample. The resultant mixture was incubated at 90 °C for 95 min and absorbance were recorded at 645 nm via UV spectrophotometer. Ascorbic acid was used as positive control and antioxidant capacity was expressed as µg AAE/mg extract [38].

#### 2.4.4. Total Reducing Power

Reducing power of *E. intermedia* aqueous extract was determined following our previously reported method [39]. Briefly, 0.5 mL of phosphate buffer having pH of 6.6 was added to 0.2 mL of plant sample, 1% potassium ferricyanide and incubated at 50 °C for 20 min. Subsequently, 0.5 mL of trichloroacetic acid was added to the mixture and centrifuged at 3000 rpm for 10 min. After centrifugation, the supernatant layer was mixed with 5 mL of ferric chloride and 0.1 mL of distilled water. Finally, absorbance was observed at 700 nm using ascorbic acid as control. Results were expressed in µg AAE/mg of extract.

#### 2.4.5. DPPH Anti-Radicals Assay

The antioxidant potentials of *E. intermedia* aqueous extract was evaluated following our previously reported method [40,41]. Briefly, from each concentration of sample solution, 20 µL was added to 96 well plates (Biotech USA, micro plate reader Elx 800) with subsequent addition of 180 µL of pre-prepared DPPH solution. The mixture was incubated at 37 °C for 1 h in a dark place and absorbance were recorded at 516 nm. Ascorbic acid was used as positive control and percent scavenging effect was calculated using formula;

% scavenging effect = 1 − Aborbance of sample/Absorbance of control × 100

### 2.5. Synthesis and Optimization of Nanoparticles

Different optimization parameters were taken under consideration during synthesis of *E. procera* nanoparticles (EpNPs). For AgNO_3_ salt concentration optimization, synthesis of EpNPs was done at different concentrations (0.5, 1, 1.5, 2, 2.5, 3 and 10 mM) of AgNO_3_. Briefly, 100 µL plant extract was taken in different Eppendorf tubes and 1 mL of each concentration of AgNO_3_ solution was added to Eppendorf tubes. For pH optimization, the same protocol was followed using different pH (5, 7, 8 and 9) of plant extracts. Ratio base optimization was done with different (Ext:AgNO_3_) ratios of (1:1, 1:2, 1:3, 1:5, 1:7, 1:10 v/v) [42]. Temperature based optimization includes synthesis of EpNPs at 30, 37, 50 and 60 °C. To find out the optimum reduction time of silver ions, absorbance intensities of reaction mixtures were measured at different time intervals (0 min–24 h).

#### Characterization of EpNPs

The UV-spectra was obtained by scanning the samples in the range between 200 and 800 nm. The functional characterization of biomolecules present in EpNPs was done by Fourier-transform infrared spectroscopy (FTIR) at frequency range of 4000–500 cm^−1^. Crystallographic structures of EpNPs were determined using X-ray diffraction (XRD), with Cu Kα as radiation source (40 kV and 30 mA) and (λ = 1.540 nm) and angular range 10° to 80. The size and morphological analysis of EpNPs was done by high-resolution scanning electron microscopy (SEM) analysis (MIRA3 TESCAN).

### 2.6. Biological Studies

#### 2.6.1. Antibacterial Studies

EpNPs were evaluated for their antibacterial potential against pathogenic bacterial strains using agar well diffusion assay [43]. Overnight cultures were used, and turbidity of each inoculum was set with standard (0.5 McFarland) solution. The refreshed inoculums (100 μL) were swabbed onto Tryptic soy agar plates. Wells on Tryptic soy agar plates were made with help of sterilized cork borer. EpNPs (30 μL) and 100 μg/mL DMSO were added to wells and the plates were incubated for 24 h at 37 °C. The clear zones of inhibition were measured with help of vernier caliper. DMSO and cefixime were used as negative and positive controls, respectively. Bacterial strains ≥10 mm DIZ were evaluated at lower concentration (1.1 to 100 μg/mL) of EpNPs for their minimum inhibitory concentrations (MICs) using broth micro dilution method [44]. Bacterial suspension of 50 μL of each strain was added to a sterile 96-well microplate containing different concentrations of EpNPs. The plates were incubated at 37 °C for 24 h. The concentration of EpNPs at which no growth was observed was taken as MIC value.

#### 2.6.2. Antifungal Studies

Disc diffusion method was used for antifungal activity of EpNPs following previously reported protocol [45,46]. The fungal strains were grown on SDA and turbidity of fungal spores suspended in 0.02% Tween 20 solution was set according to standard 0.5 McFarland solution. SDA plates were swabbed with 100 μL of harvested spores. Filter paper discs loaded with 5 μL of EpNPs (100 μg/disc) were placed on swabbed SDA plates. Discs immersed in DMSO and 5μL of amphotericin B (250 µg/mL) and were used at negative and positive controls respectively. After incubation at 28 °C for 24 h, clear zones of inhibition were measured with help of Vernier caliper. Experiments were performed in triplicate.

#### 2.6.3. HepG2 Cytotoxicity

##### Cells Culture

HepG2 cancer cell lines were cultured in DMEM (pH = 7.2) containing 10% Fetal Bovine Serum (FBS), 100 U/mL of antibiotic Gentamycin, and grown at 37 °C in a humidified atmosphere of 5% CO_2_. Confluent cells (80–90%) were harvested using trypsin after washing in phosphate-buffered saline [47].

##### MTT Cell Viability Assay

MTT assay was used to check effects of nanoparticles as well as extract on the cells viability. The assay is based on conversion of MTT dye by cellular dehydrogenases of viable cells to a blue color crystalline formazan. Cells were put in 96-wells plates (1 × 10^4^/100 µL of medium/well) and left for 24 h. EpNPs suspension (100 µL) and extract solutions (7.8, 15.62, 31.25, 62.5 125, 250 and 500 µg/mL) were added to each well and incubated for 24 h. After incubation, 10 μL of sterilized MTT solution (1 mg/mL in distilled H_2_O) was added and plate was incubated again for 4 h at 37 °C in humidified CO_2_ (5%) incubator. Thereafter, 100 µL of DMSO was added to each well and mixed with the cells thoroughly for the complete dissolution of formazan crystals. Micro plate Wells containing cells without AgNPs and an extract only were considered as negative control. The absorbance was monitored at 570 nm using an ELISA micro-plate reader. The cell viability was expressed as a percentage of the viability of the control cells [48].

### 2.7. Hemolytic Assay

Hemolytic assay was performed to check the effect of synthesized EpNPs following reported protocol [49]. Fresh human blood (1 mL) was taken in EDTA tube and centrifuged at 14,000 rpm to isolate RBCs. Thereafter, the supernatant was discarded, 200 µL from pellet (blood) was transferred to falcon tube and 9.8 mL of phosphate buffer saline (pH: 7.2) was added to it followed by centrifugation at 2000 rpm for 10 min. Further, 100 µL of RBCs suspension in PBS was placed in 96-well plate and followed by addition of 100, 50, 25, 12, and 6 µg/mL EpNPs. Plates were incubated for one h at 35 °C. Triton X-100 (0.5%) was taken as positive control while PBS and water was used as negative control. Percentage of hemolysis was calculated using formula:

$$Percent\;hemolysis=\;100\;\times \left(\frac{Abs\;of\;\;samples\;-Abs\;of\;Neg.\;\;control}{Abs\;of\;\;Pos.\;\;control-\;Abs\;of\;Neg.\;\;control\;}\right)$$

### 2.8. Statistical Analysis

All experiments were performed in triplicates; Data was presented as mean ± SD of three independent experimental observations. Origin Pro 8 and GraphPad software were used to present data graphically and calculate IC_50_ values.

The study was approved by Research Ethics Committee, Department of Biotechnology, Quaid-i-Azam University Islamabad, Pakistan, Dated 10-3-2016 via reference no: DREC/BIO/20160502/02.

## 3. Results

### 3.1. Phytochemical Analysis

The preliminary phytochemical screening of aqueous extract of aerial parts of *E. procera* confirms the presence of phenols and Flavonoids. As shown in Table 1, total flavonoids of 20.7 ± 0.21 µg/mg extract and total phenols 117.01 ± 0.78 µg/mg extract were observed. The estimated total antioxidant activity was 73.8 ± 0.32 µg/mg extract, DPPH free radical scavenging of 71.8 ± 0.73 and reducing power of 105.4 ± 0.65 µg/mg extract.

### 3.2. Characterization of EpNPs

#### 3.2.1. Uv-Visible Spectroscopy

**Figure 1:**
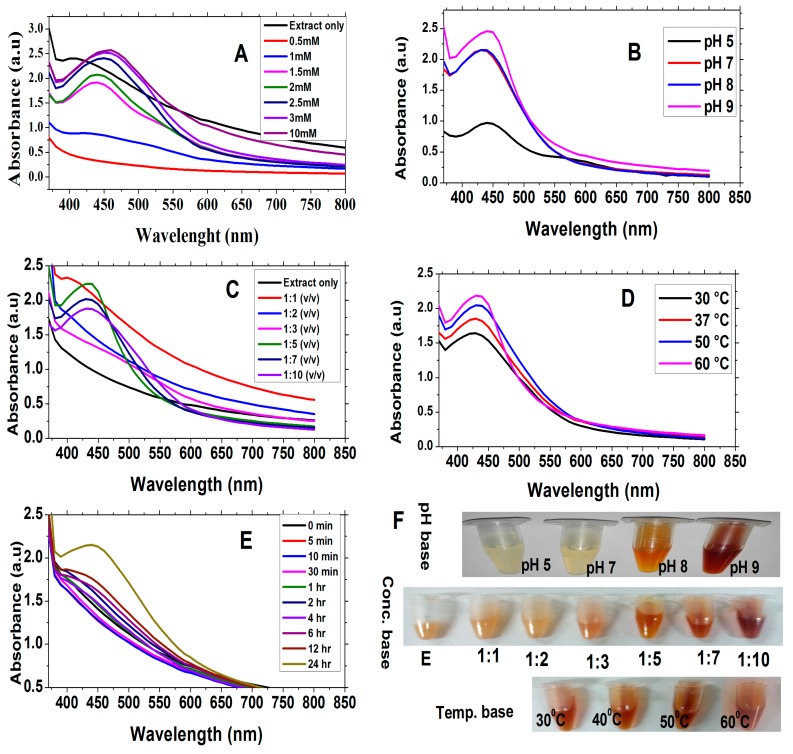
Optimization of *E. procera* nanoparticles (EpNPs) using different parameters: (**A**) Concentration of AgNO3, (**B**) pH, (**C**) Ratio of AgNO3 and Plant extract, (**D**) Temperature, (**E**) Time intervals and **(F**) Optimization images.

The color of the reaction mixture was changed which confirmed the synthesis of EpNPs. Figure 1A shows the UV-visible spectra of EpNPs at different concentration of AgNO_3_ salt. Maximum absorbance was recorded at 458 nm for 10 mM of AgNO_3_ Table 2. In pH optimization, as the pH of extract was increased, the color of reaction mixture changed from light-yellow to dark-brown which confirms the synthesis of EpNPs as shown in Figure 1B. The Uv-spectra shows that maximum surface plasmonance of 444 nm at alkaline pH 9 (Table 2). Figure 1C shows the UV spectra of EpNPs. Maximum surface plasmonance of 436 nm was observed at 1:5 (v/v) in Table 2. The increase in absorbance intensity correlates to excessive synthesis of NPs due to the reduction of silver ions. Figure 1D shows the UV-spectra of EpNPs at different temperatures. The recorded absorbance intensities of reaction mixture increased with increase in temperature which show high quantity production of EpNPs as shown in Table 2 [50]. During time optimization, it was observed that the absorbance increased with time which indicated the production of EpNPs, as shown in Figure 1E. Whereas, Figure 1F indicates the various images taken during the experimental process for optimization.

#### 3.2.2. Fourier-Transform Infrared Spectroscopy

**Figure 2:**
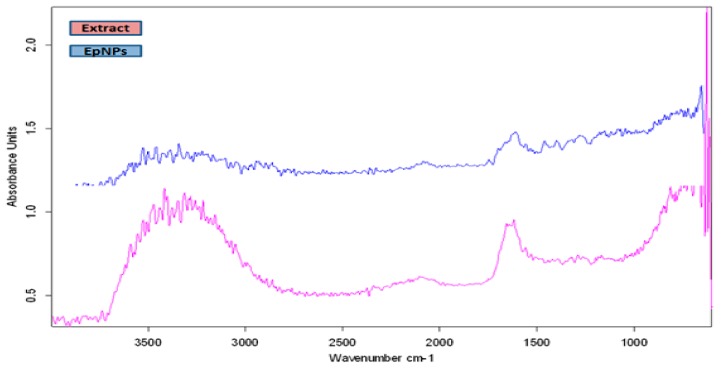
Fourier-transform infrared spectroscopy (FTIR) spectra of biosynthesized *E. procera* nanoparticles (EpNPs). A comparison of plant extract and EPNPs has been shown.

The FTIR analysis of EpNPs was done to determine the biomolecules which are involved in a capping, reduction and stabilization of EpNPs. Figure 2 shows the FTIR spectra of aqueous extract and EpPNs. The wide band in the range of 3000–3400 cm^−1^ represents ammonia and hydroxyl groups in protein molecules [51]. A broad peak at 3290 cm^−1^ shows OH stretching due to alcoholic group [52]. Peaks at 2190 and 2040cm^−1^ are arise form to CC or CN triple bond, respectively [53]. The peak at 1624 cm^−1^ represent the presence of carbonyl (C–O) group [54].

### 3.3. X-rays Differaction Analysis

**Figure 3:**
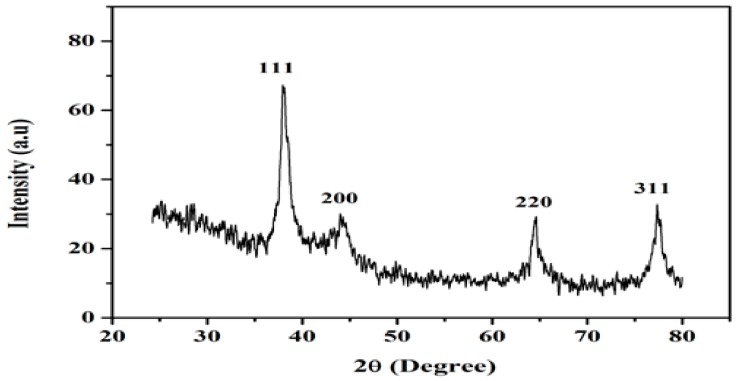
X-ray diffraction (XRD) spectra of EpNPs

EpNPs were evaluated by X-ray diffraction for their crystalline nature. The diffraction pattern indicated four main peaks at 2θ values of 38.01, 43.99, 64.42 and 77.48 corresponding to 111, 200, 220 and 311 crystallographic planes of face-centered cubic silver respectively as shown in Figure 3. Scherrer’s equation was used to calculate the mean particle size of silver nanoparticles: (*D = Kλ/β cosθ*). Where D represents the size of EpNPs, K is the Scherrer constant (values ranges from 0.9 to 1), λ represents the wavelength of the X-ray source (1.540) used in XRD, β is the FWHM (full width at half maximum of the diffraction peak), and θ is the Bragg angle. The average crystallite size of the EpNPs was 17.2 nm, derived from the full width at half maximum of peak corresponding to the (111) plane.

### 3.4. SEM Analysis of EpNPs

**Figure 4:**
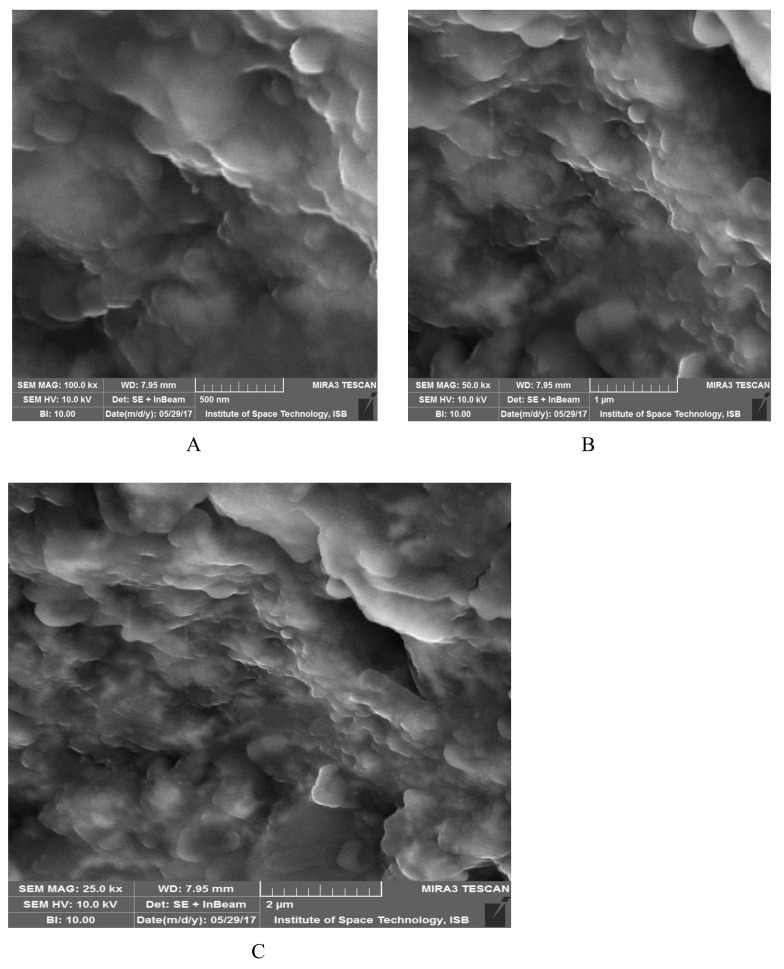
(**A**–**C**) Scanning electron microscopy (SEM) of EpNPs synthesized from aqueous extract of *Ephedra procera* plant. (**A**) Image taken at 500 nm, (**B**) Image taken at 1 µm, (**C**) Image taken at 2µm.

**Figure 5:**
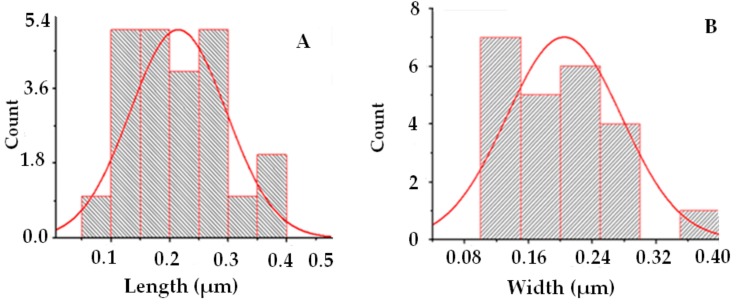
(**a**) Histogram showing average length distribution (**b**) and average width distribution.

Figure 4A–C shows the SEM results of synthesized EpNPs. The micrograph indicates that particles are of spherical shapes. Most of the NPs were aggregated as clusters, and only few of them were distributed. Figure 5a,b shows the size distribution of EpNPs. The histogram obtained reveals mono-dispersed nature of EpNPs particles. The average diameter statistically calculated is in promising similarity (20.4 nm) when compared with manually obtained calculations. The same length and width of the particles distribution further clarifies the spherical morphology of obtained sample.

### 3.5. Anti-Microbial Studies

EpNPs showed high activity against *E. coli* and *B. subtilis* with MIC of 11.33 μg/mL and 11.12 μg/mL, respectively. EpNPs showed moderate activity against *P. aeruginosa* while the *S. epidermidis* and *S. aureus* strains were found resistant. EpNPs showed considerable antifungal activity against *A. flavus* and *A. niger* while moderate activity against *Mucor* spp. (Table 3 and Table 4). EpNPs showed antifungal activity against *A. flavus*, *A. niger* and *Mucor* spp. with DIZ of 14.2 ± 1.42, 15.8 ± 1.72 and 11 ± 0.78 mm respectively.

### 3.6. Cytotoxicity Against HepG2 Cells

**Figure 6:**
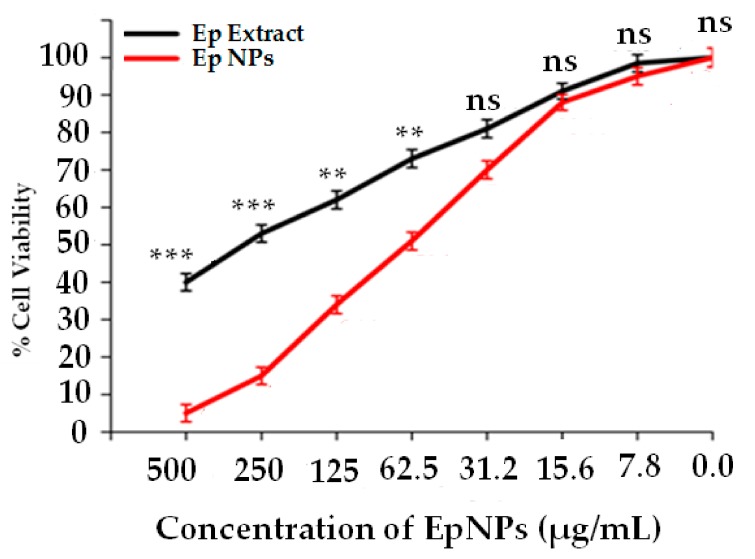
Anticancer activity of EpNPs and *Ephedra procera* extract. Values significantly different in comparison to positive control; *** *p* < 0.001, ** *p* < 0.01 and ns: values not significantly different as compared to standard drug.

Dose dependent cytotoxic effects were observed for both EpNPs and *E. procera* aqueous extract as shown in Figure 6. Median inhibitory concentrations (IC_50_) were 61.3 and 247 µg/mL for EpNPs and extract respectively.

### 3.7. Hemplytic Studies on EpNPs

**Figure 7:**
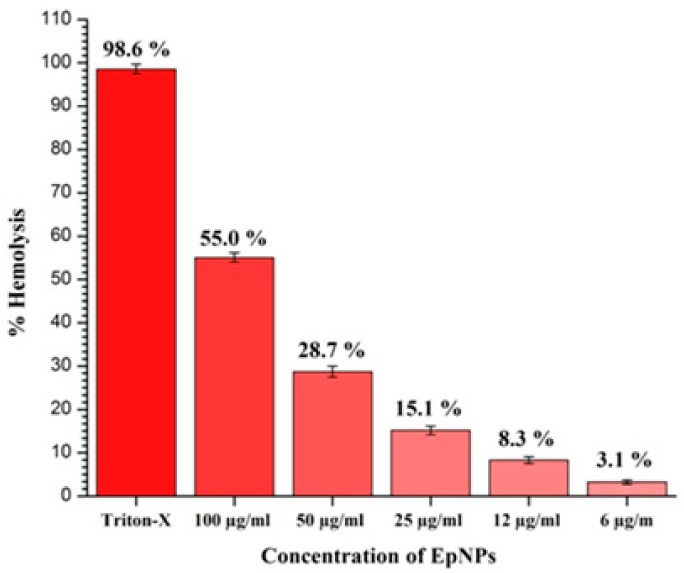
Hemolytic activity of EpNPs against human erythrocytes.

Figure 7 shows the percent hemolysis of different concentrations of EpNPs. It was observed that percent hemolysis of RBCs increases with increase of EpNPs concentration. Lowest and highest hemolysis of 3.1% and 55% were recorded at 6 and 100 µg/mL EpNPs respectively.

## 4. Discussion

Green synthesis is a recently emerging approach at the interface of medicinal plants and nanotechnology using plant extracts as reducing agents [12,13]. In the current study, we used aqueous extract *E. procera* for synthesis of silver nanoparticles and resulting EpNPs were evaluated for biological applications. Plant aqueous extract were initially evaluated for TPC, TFC, antimicrobial and antioxidant potentials [55]. Certain flavonoids and phenolic compounds are involved in scavenging of free radicals and lipid peroxidation inhibition. According to Makarov and co-workers, certain flavonoids and other biomolecules are involve as bio-reductants during synthesis of metal nanoparticles [56].

UV-Vis spectroscopy is an important characterization tool for the formation, stability and size of nanoparticles in aqueous suspensions. Reaction mixture containing AgNPs give characteristic UV peak in the range of 420–480 nm [42]. Maximum absorbance was recorded at 458 nm for 10 mM of AgNO_3._ It is already reported that higher concentration of AgNO_3_ give the maximum absorbance [50]. In optimization of NPs, pH also has a very crucial role, defining its morphology, size and biological potentials [57]. It was observed that synthesis of EpNPs increased with an increase in pH of extract. This might be due to the ionization of phenolic compounds in the extract [58]. It is reported that acidic pH results in formation of larger size particles while alkaline pH result smaller size particles [59]. Our results are supported by the previous work, whereby NPs tend to aggregate at lower pH [60]. Extract and AgNO_3_ ratio and temperature also very vital in the synthesis of NPs with desired biological properties. Our results suggest that the rate of NPs synthesis at room temperature can be enhanced by increasing temperature of the reaction mixture. While, on the other hand, the particles tend to be poly-dispersed at high temperature [61]. To find the optimum time for synthesis of AgNPs, UV analysis was done after different time interval. Initially the absorbance was increased, but after 24 h absorbance did not increased which confirm that all the AgNO_3_ salt present in the reaction mixture has been reduced by plant extract [57].

The FTIR analysis showed that the plant extract contains phytochemicals such proteins, alcohols, and carboxylic acids which are involve in reduction and capping. Extract of *E. procera* is already reported to contain certain phytochemical such as proteins, flavano-flavonol ephedrannin, Ephedroxane, Cyclopropyl-a-amino acids, Flavones, Flavanols, Bisflavanolsuch and carboxylic acids [62]. These phytochemicals are involved in AgNO_3_ and attach on the surface of silver nanoparticles. The X-ray diffraction analysis shows the average crystallite size of the EpNPs was 17.2 nm, derived from the full width at half maximum of peak corresponding to the (111) plane. Scanning electron microscopy was done to determine the size and morphology of synthesized EpNPs. The SEM results shows that EpNPs are mono-dispersed with averages diameter statistically calculated is 20.4 nm.

Antibiotics resistance is a global issue, and there is a dire need to find more useful alternatives [55] and scientists are struggling to find novel phytochemicals to fill this gap. Silver in its chemical form is reported to have antibacterial potentials [63]. The green synthesized AgNPs are reported to have high antibacterial potential against different pathogenic strains and could be used as an alternative to antibiotics [64]. In our study, we observed that EpNPs showed high activity against *E. coli* and *B. subtilis.* Marslin and colleagues have also reported that green synthesized AgNPs show good activity against *E. coli* [57]. *Cleome viscosa* L. AgNPs are reported to have dose defendant antibacterial activity [65]. Literature shows that the exact mechanism of AgNPs is not well known. According to reports, [66] AgNPs make pits in the cell wall of bacteria which contributed AgNPs for their antibacterial potential. It is reported that cell membrane of bacterial is negatively charge while AgNPs are positively charged. Thus, assembled on the membrane, this results in structural conformation of membrane and increases permeability across the membrane, which ultimately causes cell death [67]. Another study reported that AgNPs damage the genetic material of bacterial and inhabit transcription and translation [68].

The synthesized AgNPs showed good antifungal activity against test fungal strains. High and low antifungal activities were observed against *A. niger* and *Mucor* spp. receptivity. It is already reported in literature that green synthesized AgNPs possess high antifungal potential [69]. It is also reported by Medda and co-workers that AgNPs show high antifungal activity against *Aspergillus* spp. [70]. The antifungal activity of AgNPs is dependent on the size of AgNPs [71]. It is also that Ag^+^ changes these conformation and function of those proteins which are involved in cell cellular respiration [72].

Primary liver cancer (PLC) is the sixth most frequent cancer worldwide and it results second most common cause of cancer death [73]. Liver cancer causes about 700,000 deaths annually [74]. Treatment methods for liver cancer involve interventional therapy, radiofrequency ablation, surgery, microwave ablation, chemotherapy, radiation therapy, targeted therapy and liver transplantation [73]. Nano-biotechnolgy is an emerging field which covers a wide range of applications. The green synthesized AgNPs are reported to have anticancer potential against liver cancer and in the near future, it will find a way toward cancer therapeutics [75]. In our study, we observed dose dependent cytotoxic activity for both EpNPs and *E. procera* aqueous extract against liver cancer cell lines. The exact mechanism of AgNPs for the cytotoxic potential is not well known, but some reports state that AgNPs inhabit cell cycle by disrupting certain genes which are involved in the cell cycle regulation and also induces DNA damage and apoptosis in cancer cells [76]. One study reported that the cytotoxic effect of AgNPs is due to interaction of silver and certain functional groups present in intra-cellular proteins help in DNA replication [77]. It is also hypothesized that AgNPs have the ability to generate reactive oxygen species, which could result in DNA damage and lead to death of cancerous cells [54]. The release of silver ions from the colloidal nanoparticles can lead to the cytotoxicity against cancer cells. The release of silver ions is reported to be high in the acidic environments of tumor. One of the studies indicated that in an acidic environment, the release of silver ions can become doubled and thus resulted in selective killing of cancer cells [19].

The synthesized EpNPs were evaluated for their hemo-computability against human erythrocytes. It was observed that hemolysis increased when the concentration of EpNPs increased. In the study, EpNPs were found to be safe at a concentration of 6 µg/mL according to ASTM E2524-08 standard [78]. The intensity of hemolysis is proportional of the size of NPs [78].

## 5. Conclusions

Approaches of nanoparticle production through various physical and chemical ways have their own shortcomings as they produce massive environmental pollutions and lethal side effects. Therefore, the current trend of research encourages researchers all across the world to go for green synthesis of nanoparticles which is an easy, cost-effective, eco-friendly, well-controlled and non-toxic approach. Green synthesized EpNPs (17.2 nm) were found to possess high antibacterial activity against *Bacillus subtilis* and *Escherichia coli* among tested bacterial strains. *Aspergillus niger* fungal strain was found to be more sustainable against EpNPs. It was observed that synthesized EpNPs were bio-compatible and have relatively less harmful effects on human erythrocytes. It was also found that EpNPs have high cytotoxic activity against human liver cell lines (HepG2). Further studies are required for potential applications of EpNPs in various diseases.

## Figures and Tables

**Table 1:** Phytochemical analysis of *E. procera*.

S. No	Phytochemicals	µg/mg Extract	Correlation
1.	Total Flavonoids ^1^	20.7 ± 0.21	y = 0.0058x + 0.0538
			R^2^ = 0.9925
2.	Total Phenolic content ^2^	117.01 ± 0.78	y = 0.0427x + 0.1448
			R^2^ = 0.9826
3.	Antioxidant capacity ^3^	73.8 ± 0.32	y = 0.0021x + 0.099
			R^2^ = 0.9802
4.	Total reducing power ^3^	105.4 ± 0.65	y = 0.0236x + 0.0996
			R^2^ = 0.9661
5.	Free radical scavenging ^3^	71.8 ± 0.73	y = 0.3111x − 0.2215
			R^2^ = 0.9805

All the values given are means of ± standard error of three separate experiments. Data presented as the mean ± standard deviation. ^1^ Quercetin equivalent, ^2^ Gallic acid equivalent, ^3^ Ascorbic acid equivalent.

**Table 2:** UV-Absorbance peaks at different optimization parameters.

Concentration Based		Reaction pH Based		Reaction Temperature Based		Ratio Based	
Conc. of AgNO_3_ (mM)	Peaks	pH of Reaction Mixture	Peaks	Temperature of Reaction Mixture	Peaks	Ratio of Extract and AgNO_3_ (*v*/*v*)	Peaks
0.5	661	5	442	30 °C	427	1:1	400
1	420	7	435	37 °C	431	1:2	Np
1.5	438	8	436	50 °C	436	1:3	Np
2	442	9	444	60 °C	436	1:5	436
2.5	449					1:7	434
3	455					1:10	436
10	458						

Np: No peak, mM: Millimolar, Ext: Extract, nm: Nanometer.

**Table 3:** Antibacterial activities of EpNPs.

Antibacterial Activity of EpNPs			
Bacterial Strains	DIZ (mm) EpNPs	MICs μg/mL	DIZ (mm) CF
*B. subtilis* (ATCC-6633)	15.2 ± 1.12 ***	11.33	20 ± 1.33
*P. aeruginosa* (ATCC-9721)	11 ± 1.30 ^ns^	100	13 ± 1.23
*E. coli* (ATCC-25922)	19.2 ± 1.22 ***	11.12	28 ± 1.07
*S. epidermidis* (ATCC-12228)	–	–	13 ± 0.91
*K. pneumoniae* (ATCC-1705)	14.2 ± 1.74 ^ns^	33.3	18 ± 1.12
*S. aureus* (ATCC-6538)	–	–	15 ± 0.77

EpNPs: *Ephedra procera* nanoparticles, DIZ: Diameter of Inhibitory zone, CF: Cefixime, –: No activity in antibacterial assay or not active (zone ˂ 10 mm). Values significantly different in comparison to standard drug Cefixime; *** *p* < 0.001, ns: values not significantly different as compared to standard drug.

**Table 4:** Antifungal potentials of EpNPs.

Antifungal Activity		
Fungal Strains	DIZ (mm) EpNPs	DIZ (mm) Amp
*Fumigatus*	13 ± 2.03 ***	22± 1.00
*Flavus*	14.2 ± 1.42 ***	23 ± 0.89
*Niger*	15.8 ± 1.72 **	20 ± 1.09
*Mucor* spp.	11 ± 0.78 ***	22 ± 0.67

EpNPs: *Ephedra procera* nanoparticles, DIZ: Diameter of Inhibitory zone, Amp: Amphotericin B. Values significantly different in comparison to standard drug Amp; *** *p* < 0.001, ** *p* < 0.01.

## Abbreviations

TPC: Total Phenolic contents, EpNPs: *Ephedra procera* nanoparticles, TFC: Total Flavonoid contents, MICs: Minimum Inhibitory Concentration, FTIR: Fourier-transform infrared spectroscopy, SEM: Scanning electron microscope, DIZ: Diameter of Inhibitory zone, HepG2: Hepatoma G2 cell line, MTT: 3-(4,5-Dimethylthiazol-2-Yl)-2,5-Diphenyltetrazolium Bromide, ATCC: American type culture collection, XRD: X-ray diffraction, SDA: Sabouraud Dextrose agar, FBS: Fetal Bovine Serum.
